# A new investigation on fractionalized modeling of human liver

**DOI:** 10.1038/s41598-024-51430-y

**Published:** 2024-01-18

**Authors:** Sanjay Bhatter, Kamlesh Jangid, Shyamsunder Kumawat, Dumitru Baleanu, Sunil Dutt Purohit, Daya Lal Suthar

**Affiliations:** 1 Department of Mathematics, Malaviya National Institute of Technology Jaipur, Jaipur, India; 2 Department of Mathematics, Central University of Rajasthan, Ajmer, India; 3 Department of Computer Science and Mathematics, Lebanese American University, Beirut, Lebanon; 4 Institute of Space Sciences, Magurele-Bucharest, Romania; 5 Department of HEAS (Mathematics), Rajasthan Technical University, Kota, India; 6 Department of Mathematics, Wollo University, P.O. Box 1145, Dessie, Ethiopia

**Keywords:** Diseases, Mathematics and computing

## Abstract

This study focuses on improving the accuracy of assessing liver damage and early detection for improved treatment strategies. In this study, we examine the human liver using a modified Atangana-Baleanu fractional derivative based on the mathematical model to understand and predict the behavior of the human liver. The iteration method and fixed-point theory are used to investigate the presence of a unique solution in the new model. Furthermore, the homotopy analysis transform method, whose convergence is also examined, implements the mathematical model. Finally, numerical testing is performed to demonstrate the findings better. According to real clinical data comparison, the new fractional model outperforms the classical integer-order model with coherent temporal derivatives.

## Introduction

Mathematical models of various diseases have caught the interest of many scientists among the outstanding studies that already exist in this field. This area of research attracts to mathematicians and biologists more because of mathematical modelling. Fractional calculus is now frequently utilized to model and explain biological processes. Recent years have seen a rise in the modelling and explanation of biological processes using fractional calculus^1–5^.

Fractional calculus has recently grown in significance and popularity for real-world modeling issues^6–9^. However, numerous researchers have demonstrated that the standard form of fractional derivatives with a singular kernel doesn’t always adequately classify the nonlocal dynamics adequately; as a result, new kinds of fractional operators with the nonsingular kernel must be applied in order to improve and scrutinize the actual world happenings with the genetically inherited property. A novel differential operator with a Mittag-Leffler (ML) kernel (MABC) has just been created^10^ for this purpose. To successfully solve the MABC (Modified Atangana-Baleanu fractional derivative in Caputo sence) fractional models, improved numerical methods must also be developed.

The fractional derivatives are widely used to study the memory effect in the complex processes described by kernels (singular and non-singular)^11–15^. In this work, a modification of the Atangana-Baleanu fractional derivative is used, which includes essential properties related to the proper reconstruction of the initial value conditions, which, in many cases, is reproducible with the original version of Atangana-Baleanu impossible.

The advantage of this new fractional derivative in satisfying the initial conditions of the fractional derivative plays a central role in implementing various perturbative analytical methods, such as the homotopy analysis method. This method is applied to solve fractional differential equations.

The MABC fractional derivative is chosen to increase the representative power of our model, allowing greater accuracy and precision, reflecting the complex dynamics inherent in liver function. As a result, the MABC fractional operator provides more adaptable models that can be applied to study real-world phenomena and understand their complex dynamics.

This encourages us to investigate the features of the human liver, utilizing its novel fractional MABC calculus formulation. We also create an effective numerical method based on the iterative approach to answer the aforementioned equations correctly. According to simulation studies, using a nonsingular operator can reveal model characteristics that are otherwise concealed when using the model in a traditional integer way. As a result, non-integer calculus provides more adaptable models that may be applied to study actual world happenings and comprehend their intricate dynamics.

According to^16^, the liver is a typically triangular organ that spans the whole abdominal cavity beneath the diaphragm. Bromsulphthalein (BSP) is a colouring agent; after being injected into the blood, BSP is eliminated from the body through the liver because no other organs can take it up. Physiologists have used it for many years to investigate liver function. At various periods, the blood’s BSP concentration is assessed. From a practical standpoint, this approach is rather straightforward; it provides a continuous sequence of numbers illustrating the more or less rapid decline of BSP in the blood, which is used to investigate the function of the liver.

In healthy humans, up to 80$\%$ of BSP is dissolved in the blood in the first 25 minutes; the remaining amount is wholly dissolved in the next 20 minutes. Čelechovská^17^, Calvetti et al.^18^, Bonfiglio et al.^19^, Dumitru et al.^20^, Friedman and Hao^21^, have all made notable efforts in this approach.

This manuscript suggests a novel human liver model using the MABC operator and ML kernel of the fractional derivative resulting from the above discussion. To align with the natural biological context, the non-integer order derivative is employed in the Caputo sense rather than the Riemann-Liouville one. A parameter adjustment is also applied to avoid dimensional mismatch in the derived fractional equations. This integrated approach aims to provide a more comprehensive and biologically relevant representation of the human liver system.

The FPT (fixed point theory) also looks into the possibility of a singular solution. A novel and simple numerical approach are suggested to simulate the resultant model properly. A comparison is made between clinical data obtained by Čelechovská^17^ in 1985 and data obtained from a fractionalized model study by Dumitru et al.^20^ in 2020 and the expected values obtained by the model. Comparative outcomes demonstrate that the proposed fractional model is more effective than the traditional model that already existed.

The upcoming sections of this manuscript are organised as follows. In Section "Essential definitions", some preliminary findings are presented. The new fractional human liver model and its key features are covered in Section "Formulation of the model". Section "Presence of a unique solution" suggests a generalized numerical approach for resolving the MABC Human Liver Model. Section "Stability analysis by FPT" offers numerical and comparative information. Section "Evaluation of the iteration method’s stability" brings the paper to a close with a few closing remarks.

## Essential definitions

Some of the major mathematical definitions utilised in this work are as follows:

### Definition 2.1

The following Mittag-Leffler functions, $E_{\rho }({\mathscr {G}})$ and $E_{\rho , \tau }({\mathscr {G}})$, were introduced by Mittag-Leffler^22^ and Wiman^23^, respectively.

$$E_{\rho } ({\mathscr{G}}) = \sum\limits_{{k = 0}}^{\infty } {\frac{{{\mathscr{G}}^{k} }}{{\Gamma (\rho k + 1)}}} ;({\mathscr{G}},\rho \in \mathbb{C},\Re(\rho ) > 0),$$

and

$$E_{{\rho ,\tau }} ({\mathscr{G}}) = \sum\limits_{{k = 0}}^{\infty } {\frac{{{\mathscr{G}}^{k} }}{{\Gamma (\rho k + \tau )}}} ;\left( {{\mathscr{G}},\rho ,\tau \in \mathbb{C},\Re (\rho ) > 0,\Re (\tau ) > 0} \right).$$

### Definition 2.2

The standard definition of the Riemann-Liouville fractional integral^24^ of *h* of order $\Re (\nu )>0$ is given as follows:

$$\begin{aligned} {}_{b}{\mathbb {I}}^{\nu }_{y} h(y)= \frac{1}{\Gamma (\nu )} \int _{b}^{y} (y-\xi )^{\nu -1} h(\xi ) d\xi . \end{aligned}$$

### Definition 2.3

Let *g* be a real-valued picewise continuous function $(0, \infty )$. The Laplace transform (LT) of *g*(*z*)^25^ of exponential order $\alpha >0$ concerning *z* is given as follows;

$$\begin{aligned} {\mathscr {L}}[g(z);s] = {\bar{g}}(s) ={\mathscr {L}}[g(z)](s) = \int _{0}^{\infty }e^{-sz}g(z)dz, \quad \Re {(s)}>\alpha , z\ge 0. \end{aligned}$$

### Definition 2.4

For the function ${\bar{g}}(s)$, the inverse Laplace transform with respect to $y\ge 0$ is given as follows^25^;

$$\begin{aligned} {\mathscr {L}}^{-1}[{\bar{g}}(s);y] = g(y) = \frac{1}{2\pi i} \int _{\varGamma -i\infty }^{\varGamma +i\infty } e^{sy}{\bar{g}}(s)ds, \end{aligned}$$

here $\varGamma \in {\mathbb {R}}$ is a constant.

### Definition 2.5

The standard definition of the MABC^10^ of *f* of order $\vartheta \in \ (0,1)$ is given as follows:

$$\begin{aligned} {}^{{\textit{MABC}}}{}_{0}{\mathscr {D}}^{\vartheta }_{t}[f(t)]=\frac{M(\vartheta )}{1-\vartheta } \left [ f(t)-E_{\vartheta }(-\mu _{\vartheta } t^{\vartheta }) f(0) - \mu _{\vartheta } \int _{0}^{t} (t-\xi )^{\vartheta -1} E_{\vartheta , \vartheta } \left (-\mu _{\vartheta }(t-\xi )^{\vartheta }\right ) f(\xi ) d\xi \right ], \end{aligned}$$

where $$\mu _{\vartheta }=\frac{\vartheta }{1-\vartheta }$$, and $$M(0)=1=M(1)$$.

### Definition 2.6

The novel fractional derivative with a non-local kernel associated with the MAB fractional integral^10^ is defined as follows:

$$\begin{aligned} {}^{{\textit{MAB}}}_{0}{\mathbb {I}}^{\vartheta }_{t}[f(t)]= \frac{1-\vartheta }{M(\vartheta )} \left [ f(t) + \mu _{\vartheta } \ {}^{RL}_{0}{\mathbb {I}}^{\vartheta }_{t}[f(t)] -f(0) - \mu _{\vartheta } f(0) \frac{t^{\vartheta }}{\Gamma (\vartheta +1)} \right ]. \end{aligned}$$

### Definition 2.7

The LT of MABC is presented as follows^10^:

$$\begin{aligned} {\mathscr {L}}[{}^{{\textit{MABC}}}_{0}{\mathscr {D}}^{\vartheta }_{t} f(t); {\textsf{P}}]= \frac{M(\vartheta )}{1-\alpha } \ \frac{{\textsf{P}}^{\vartheta } [f(t); {\textsf{P}}]-{\textsf{P}}^{\vartheta -1} f(0)}{{\textsf{P}}^{\vartheta }+\mu _{\vartheta }}. \end{aligned}$$

## Formulation of the model

**Figure 1:**
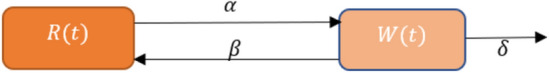
Simple process of BSP administration through blood and liver.

Čelechovská^17^ proposed a model of the human liver with integer order in 2004. For parameter identification within that study, the author employed the clinical information collected by the BSP test. Let *R*(*t*) and *W*(*t*) represent the concentrations of BSP in blood and liver at time *t*, respectively; the flow transfer of BSP between them is shown in Figure 1. Then, the integer-order model suggested by Čelechovská is described as follows:

1 $$\begin{aligned} \left. \begin{aligned}{}&\dfrac{dR(t)}{dt}=-\alpha R(t) + \beta W(t), \\&\dfrac{dW(t)}{dt}= \alpha R(t) - (\beta +\delta ) W(t). \ \ \end{aligned} \hspace{1cm} \right\} \end{aligned}$$

Where $R(0)=R_{0}$ and $W(0)=W_{0}$ are initial conditions ($R_{0}>0$), and the transfer rates are represented by known parameters $\alpha , \beta , \delta$.

We stabilize the system by replacing the time derivative with the MABC^10^. The right, as well as the left sides, will no longer be the same dimension as with this alteration. To resolve this issue, we adjust the fractional operator such that both sides have an equal dimension by using an additional parameter with the dimension of time called $\rho$^26^. The reasoning provided leads to the following explanation of the Human liver fractional model for $t\ge 0$ and $\vartheta \in (0,1)$

2 $$\begin{aligned} \left. \begin{aligned}{}&\frac{1}{\rho ^{1-\vartheta }}\ {}^{{\textit{MABC}}}{\mathscr {D}}_{t}^{\vartheta } R(t)=-\alpha R(t)+ \beta W(t), \\&\frac{1}{\rho ^{1-\vartheta }}\ {}^{{\textit{MABC}}}{\mathscr {D}}_{t}^{\vartheta } W(t)= \alpha R(t)-(\beta +\delta )W(t). \end{aligned} \hspace{1.5cm} \right\} \end{aligned}$$

The next section investigates whether the system (2) solution to the FPT exists and is unique.

## Presence of a unique solution

In this part, we demonstrate that the system has a singular solution. To do this, we use Nieto and Losada’s^27^ fractional integral operator on the system (2), and we get

$$\begin{aligned} \begin{aligned}{}&R(t)-R(0)= \rho ^{1-\vartheta }\ {}^{{\textit{MABC}}}{\mathscr {D}}_{t}^{\vartheta } [-\alpha R(t)+ \beta W(t)], \\&W(t)-W(0)= \rho ^{1-\vartheta }\ {}^{{\textit{MABC}}}{\mathscr {D}}_{t}^{\vartheta } [ \alpha R(t)-(\beta +\delta )W(t)]. \ \ \end{aligned} \end{aligned}$$

Using the definition of MABFI^10^, we have

3 $$\left. \begin{aligned} R(t) - R(0) = &\, \rho ^{{1 - \vartheta }} \frac{{(1 - \vartheta )}}{{M(\vartheta )}}[ - \alpha R(t) + \beta W(t) + \mu _{\vartheta } ^{{RL}} {\mathscr{D}}_{0}^{\vartheta } ( - \alpha R(t) + \beta W(t)) \hfill \\& - ( - \alpha R(0) + \beta W(0))(1 + \mu _{\vartheta } \frac{{t^{\vartheta } }}{{\Gamma (\vartheta + 1)}})], \hfill \\ W(t) - W(0) =&\, \rho ^{{1 - \vartheta }} \frac{{(1 - \vartheta )}}{{M(\vartheta )}}[\alpha R(t) - (\beta + \delta )W(t) + \mu _{\vartheta } ^{{RL}} {\mathscr{D}}_{0}^{\vartheta } (\alpha R(t) - (\beta + \delta )W(t)) \hfill \\& - (1 + \mu _{\vartheta } \frac{{t^{\vartheta } }}{{\Gamma (\vartheta + 1)}})(\alpha R(0) - (\beta + \delta )W(0))] \hfill \\ \end{aligned} \right\}$$

For convenience, we consider

$$\begin{aligned} q_{1}(t, R)=&-\alpha R(t)+\beta W(t) ,\\ q_{2}(t, W)=&\alpha R(t)-(\beta +\delta )W(t). \end{aligned}$$

### Theorem 4.1

The Lipschitz condition (LC) and contraction are fulfilled by the kernel $q_{1}$ if the following disparity persists: $0<\alpha \le 1$.

### Proof

Consider the function *R*(*t*) and $R_{1}(t)$, then

$$\begin{aligned}{}&\Vert q_{1}(t, R(t))-q_{1}(t, R_{1}(t))\Vert \\&=\Vert -\alpha R(t)+\beta W(t)+\alpha R_{1}(t)-\beta W(t)\Vert \le \alpha \Vert R(t)-R_{1}(t)\Vert \end{aligned}$$

Thus, the LC is fulfilled for $q_{1}$. Additionally, if $0<\alpha \le 1$, then $q_{1}$ is a contraction. $\square$

Similarly, $q_{2}$ satisfy the LC as follows:

$$\begin{aligned} \Vert q_{2}(t, W(t))-q_{2}(t, W_{1}(t))\Vert \le (\beta +\delta )\Vert W(t)-W_{1}(t)\Vert . \end{aligned}$$

With $q_{1}, q_{2}$, in mind, the Eq. (3) may be written as follows:

$$\begin{aligned} R(t) = & R(0) + \frac{{\rho ^{{1 - \vartheta }} (1 - \vartheta )}}{{M(\vartheta )}}[q_{1} (t,R(t)) + \mu _{\vartheta } ^{{RL}} {\mathbb{I}}_{0}^{\vartheta } [q_{1} (t,R(t))] \\ &- \left( {1 + \mu _{\vartheta } \frac{{t^{\vartheta } }}{{\Gamma (\vartheta + 1)}}} \right)\left( { - \alpha R(0) + \beta W(0)} \right)], \\ W(t) = & W(0) + \frac{{\rho ^{{1 - \vartheta }} (1 - \vartheta )}}{{M(\vartheta )}}[q_{2} (t,W(t)) + \mu _{\vartheta } ^{{RL}} {\mathbb{I}}_{0}^{\vartheta } [q_{2} (t,W(t))] \\ &- \left( {1 + \mu _{\vartheta } \;\frac{{t^{\vartheta } }}{{\Gamma (\vartheta + 1)}}} \right)\left( {\alpha R(0) - (\beta + \delta )W(0)} \right)]. \\ \end{aligned}$$

Thus, consider the below recursive formula:

$$\begin{aligned} R_{n}(t)=&\frac{\rho ^{1-\vartheta }(1-\vartheta )}{M(\vartheta )} \bigg [q_{1}(t, R_{n-1}(t)) + \mu _{\vartheta } \ {}^{RL}{\mathbb {I}}^{\vartheta }_{0}[q_{1}(t, R_{n-1}(t))] \nonumber \\ &- \Big (1+\mu _{\vartheta } \ \frac{t^{\vartheta }}{\Gamma (\vartheta +1)}\Big ) \big (-\alpha R(0) + \beta W(0)\big ) \bigg ] , \\ W_{n}(t)=&\frac{\rho ^{1-\vartheta }(1-\vartheta )}{M(\vartheta )} \bigg [q_{2}(t, W_{n-1}(t)) + \mu _{\vartheta } \ {}^{RL}{\mathbb {I}}^{\vartheta }_{0}[q_{2}(t, W_{n-1}(t))] \nonumber \\ &- \Big (1+\mu _{\vartheta } \ \frac{t^{\vartheta }}{\Gamma (\vartheta +1)}\Big ) \big (\alpha R(0) - (\beta +\delta ) W(0)\big ) \bigg ], \end{aligned}$$

where $$R_{0}(t)=R(0),\ W_{0}(t)=W(0)$$.

Now, we examine

$$\begin{aligned} L_{{1n}} = & R_{n} (t) - R_{{n - 1}} (t) \\ = & \frac{{\rho ^{{1 - \vartheta }} (1 - \vartheta )}}{{M(\vartheta )}}[q_{1} (t,R_{{n - 1}} (t)) - q_{1} (t,R_{{n - 2}} (t))] \\ & + \frac{{\vartheta \rho ^{{1 - \vartheta }} }}{{M(\vartheta )}} {}^{{RL}}{\mathbb{I}}_{0}^{\vartheta } [q_{1} (t,R_{{n - 1}} (t)) - q_{1} (t,R_{{n - 2}} (t))], \\ \end{aligned}$$

and

$$\begin{aligned} L_{{2n}} = & W_{n} (t) - W_{{n - 1}} (t) \\ = & \frac{{\rho ^{{1 - \vartheta }} (1 - \vartheta )}}{{M(\vartheta )}}[q_{2} (t,W_{{n - 1}} (t)) - q_{2} (t,W_{{n - 2}} (t))] \\ & + \frac{{\vartheta \rho ^{{1 - \vartheta }} }}{{M(\vartheta )}}\;^{{RL}} {\mathbb{I}}_{t}^{\vartheta } [q_{2} (t,W_{{n - 1}} (t)) - q_{2} (t,W_{{n - 2}} (t))]. \\ \end{aligned}$$

Using the aforementioned equations, one may write

4 $$\begin{aligned} R_{n}(t)=\sum _{j=0}^{n} L_{1j}(t), \ \ \ \ \ \ \ W_{n}(t)=\sum _{j=0}^{n} L_{2j}(t). \end{aligned}$$

Using the triangular inequality and the $L_{1n}$ definition, we obtain

$$\begin{aligned} \left\| {L_{{1n}} (t)} \right\| = & \left\| {R_{n} (t) - R_{{n - 1}} (t)} \right\| \\ = & \left\| {\frac{{\rho ^{{1 - \vartheta }} (1 - \vartheta )}}{{M(\vartheta )}}\left[ {q_{1} (t,R_{{n - 1}} (t)) - q_{1} (t,R_{{n - 2}} (t))} \right]} \right. \\ & \left. { + ^{{RL}} {\mathbb{I}}_{0}^{\vartheta } \left[ {q_{1} (t,R_{{n - 1}} (t)) - q_{1} (t,R_{{n - 2}} (t))} \right]\frac{{\vartheta \rho ^{{1 - \vartheta }} }}{{M(\vartheta )}}} \right\|{\mkern 1mu} \\ \le & \frac{{\rho ^{{1 - \vartheta }} (1 - \vartheta )}}{{M(\vartheta )}}\left\| {\left[ {q_{1} (t,R_{{n - 1}} (t)) - q_{1} (t,R_{{n - 2}} (t))} \right]} \right\| \\ & + \left\| {^{{RL}} {\mathbb{I}}_{0}^{\vartheta } \left[ {q_{1} (t,R_{{n - 1}} (t)) - q_{1} (t,R_{{n - 2}} (t))} \right]} \right\|\frac{{\vartheta \rho ^{{1 - \vartheta }} }}{{M(\vartheta )}}. \\ \end{aligned}$$

$q_{1}$ satisfies the LC, therefore

$$\begin{aligned}{}&\Vert R_{n}(t)-R_{n-1}(t)\Vert \le \frac{\rho ^{1-\vartheta }(1-\vartheta )}{M(\vartheta )} \ \alpha \ \big \Vert [ R_{n-1}(t)- R_{n-2}(t)] \big \Vert \\&+ \alpha \ \frac{\vartheta \rho ^{1-\vartheta }}{M(\vartheta )} \ {}^{RL}{\mathbb {I}}^{\vartheta }_{0} \big \Vert [q_{1}(t, R_{n-1}(t))-q_{1}(t, R_{n-2}(t))] \big \Vert . \end{aligned}$$

Thus we get

5 $$\begin{aligned} \Vert L_{1n}(t)\Vert \le \frac{\rho ^{1-\vartheta }(1-\vartheta )}{M(\vartheta )} \ \alpha \ \big \Vert L_{1(n-1)}(t) \big \Vert +\frac{\vartheta \rho ^{1-\vartheta }}{M(\vartheta )} \ \alpha \ {}^{RL}{\mathbb {I}}^{\vartheta }_{0} \big \Vert L_{1(n-1)}(t) \big \Vert . \end{aligned}$$

It can be shown that similar results are obtained for $L_{2n}$ as follows:

6 $$\begin{aligned} \Vert L_{2n}(t)\Vert \le \frac{\rho ^{1-\vartheta }(1-\vartheta )}{M(\vartheta )} (\beta +\delta ) \big \Vert L_{2(n-1)}(t) \big \Vert +\frac{\vartheta \rho ^{1-\vartheta }}{M(\vartheta )} (\beta +\delta ) \ {}^{RL}{\mathbb {I}}^{\vartheta }_{0} \big \Vert L_{2(n-1)}(t) \big \Vert . \end{aligned}$$

The aforementioned result demonstrates that the system (2) has a solution.

### Theorem 4.2

The Human-Liver model (2) has a system of solutions if any occur $t_{1}, t_{2}$ such that

$$\begin{aligned} \frac{\rho ^{1-\vartheta } (1-\vartheta )}{M(\vartheta )} \lambda _{i}+\frac{\vartheta \, \rho ^{1-\vartheta }}{M(\vartheta )} \lambda _{i}t \, \le 1, \end{aligned}$$

where $$\lambda _{1}=\alpha ,\ \lambda _{2}=\beta +\delta$$.

### Proof

Assume that *R*(*t*), *W*(*t*) are bounded function. We have demonstrated that kernels $L_{in},\ i=1, 2$ satisfy the LC. Using the outcome of (5) and (6) together with the recursive technique, we obtain

$$\begin{aligned} \Vert L_{1n}(t)\Vert \le \Vert R(t)\Vert \bigg [\frac{\rho ^{1-\vartheta } (1-\vartheta )}{M(\vartheta )} \lambda _{1} + \frac{\vartheta \, \rho ^{1-\vartheta }}{M(\vartheta )} \lambda _{1} \, t \bigg ]^{n}, \\ \Vert L_{2n}(t)\Vert \le \Vert W(t)\Vert \bigg [\frac{\rho ^{1-\vartheta } (1-\vartheta )}{M(\vartheta )} \lambda _{2}+\frac{ \vartheta \, \rho ^{1-\vartheta }}{M(\vartheta )} \lambda _{2} \, t \bigg ]^{n}. \end{aligned}$$

As a result, smooth functions (4) exist. We assert that the aforementioned operations are the system’s (2) solutions. To back up this assertion, we suppose

$$\begin{aligned} R(t)-R(0)=L_{1n}(t)-X_{1n}(t), \\ W(t)-W(0)=L_{2n}(t)-X_{2n}(t). \end{aligned}$$

We get

$$\begin{aligned}{}&\Vert X_{1n}(t)\Vert = \Big \Vert \frac{\rho ^{1-\vartheta }(1-\vartheta )}{M(\vartheta )} \big [q_{1}(t, R(t))-q_{1}(t, R_{n-1}(t)) \big ] \\&+ {}^{RL}{\mathbb {I}}^{\vartheta }_{0} \big [q_{1}(t, R(t))-q_{1}(t, R_{n-1}(t)) \big ] \, \frac{\vartheta \rho ^{1-\vartheta }}{M(\vartheta )} \,\Big \Vert \, \\&\le \frac{\rho ^{1-\vartheta }(1-\vartheta )}{M(\vartheta )} \, \big \Vert \big [q_{1}(t, R(t))-q_{1}(t, R_{n-1}(t)) \big ] \big \Vert \\&+\frac{\vartheta \rho ^{1-\vartheta }}{M(\vartheta )} \, {}^{RL}{\mathbb {I}}^{\vartheta }_{0} \big \Vert \big [q_{1}(t, R)-q_{1}(t, R_{n-1}) \big ]\big \Vert \\&\le \frac{\rho ^{1-\vartheta }(1-\vartheta )}{M(\vartheta )} \lambda _{1} \big \Vert R(t)-R_{n-1}(t) \big \Vert +\frac{\vartheta \rho ^{1-\vartheta }}{M(\vartheta )} \lambda _{1} \, t \big \Vert R(t)- R_{n-1}(t) \big \Vert . \end{aligned}$$

We get by continuing this process

$$\begin{aligned} \Vert X_{1n}(t)\Vert \le \bigg [\frac{\rho ^{1-\vartheta }(1-\vartheta )}{M(\vartheta )} +\frac{\vartheta \rho ^{1-\vartheta }}{M(\vartheta )} t\bigg ]^{n+1} p \lambda _{1}^{n+1}. \end{aligned}$$

Using the recent equation’s limit as $n \rightarrow \infty$, we obtain $\Vert X_{1n}(t)\Vert \rightarrow 0$. In the same manner, we obtain $\Vert X_{2n}(t)\Vert \rightarrow 0$ and this proof is finished. $\square$

To demonstrate the solution’s uniqueness, we presume that the system (2) has an alternative solution, such as $R_{1}, W_{1}$. Then

$$\begin{aligned}{}&\Vert R(t)-R_{1}(t)\Vert = \Big \Vert \frac{\rho ^{1-\vartheta }(1-\vartheta )}{M(\vartheta )} \big [q_{1}(t, R(t))-q_{1}(t, R_{1}(t)) \big ] \\&+ {}^{RL}{\mathbb {I}}^{\vartheta }_{0} \big [q_{1}(t, R(t))-q_{1}(t, R_{1}(t)) \big ] \, \frac{\vartheta \rho ^{1-\vartheta }}{M(\vartheta )} \, \Big \Vert \, \\&\le \frac{\rho ^{1-\vartheta }(1-\vartheta )}{M(\vartheta )} \big \Vert \big [q_{1}(t, R(t))-q_{1}(t, R_{1}(t)) \big ] \big \Vert \\&+\frac{\vartheta \rho ^{1-\vartheta }}{M(\vartheta )} \, {}^{RL}{\mathbb {I}}^{\vartheta }_{0} \big \Vert \big [q_{1}(t, R(t))-q_{1}(t, R_{1}(t)) \big ]\big \Vert . \end{aligned}$$

We obtain, according to the LC of *R*

$$\begin{aligned} \Vert R(t)-R_{1}(t)\Vert \le \frac{\rho ^{1-\vartheta }(1-\vartheta )}{M(\vartheta )} \lambda _{1} \big \Vert R(t)-R_{1}(t) \big \Vert +\frac{\vartheta \rho ^{1-\vartheta }}{M(\vartheta )} \lambda _{1} t \big \Vert R(t)- R_{1}(t) \big \Vert . \end{aligned}$$

Thus

7 $$\begin{aligned} \Vert R(t)-R_{1}(t)\Vert \Bigg (1-\frac{\rho ^{1-\vartheta }(1-\vartheta )}{M(\vartheta )} \lambda _{1} -\frac{\vartheta \rho ^{1-\vartheta }}{M(\vartheta )} \lambda _{1} t \Bigg )\le 0. \end{aligned}$$

### Theorem 4.3

The Human-Liver model (2) solution is unique if the following requirements are satisfied:

8 $$\begin{aligned} \Bigg (1-\frac{\rho ^{1-\vartheta }(1-\vartheta )}{M(\vartheta )} \lambda _{1} -\frac{\vartheta \rho ^{1-\vartheta }}{M(\vartheta )} \lambda _{1} t \Bigg )\ge 0. \end{aligned}$$

### Proof

We may deduce from the condition (8) and Eq. (7) that

$$\begin{aligned} \Vert R(t)-R_{1}(t)\Vert \Bigg (1-\frac{\rho ^{1-\vartheta }(1-\vartheta )}{M(\vartheta )} \lambda _{1} -\frac{\vartheta \rho ^{1-\vartheta }}{M(\vartheta )} \lambda _{1} t \Bigg )= 0. \end{aligned}$$

So $\Vert R(t)-R_{1}(t)\Vert =0$, then $R(t)=R_{1}(t)$. Similarly, we may demonstrate that $W(t)=W_{1}(t)$.

The proof is finished. $\square$

## Stability analysis by FPT

We find a particular solution to the Human-Liver model using the Laplace transform, and then we use FPT to demonstrate the iterative method’s stability. We begin by utilizing the LT to both sides of the equations in the model (2), then

$$\begin{aligned}{}&{\mathscr {L}} \Big [\frac{1}{\rho ^{1-\vartheta }} \, {}^{{\textit{MABC}}}D_{t}^{\vartheta } \ R(t)\Big ]({\textsf{P}})= {\mathscr {L}}[-\alpha R(t)+\beta W(t)]({\textsf{P}}), \\&{\mathscr {L}} \Big [\frac{1}{\rho ^{1-\vartheta }} \, {}^{{\textit{MABC}}}D_{t}^{\vartheta } \ W(t)\Big ]({\textsf{P}})= {\mathscr {L}}[\alpha R(t)-(\beta +\delta ) W(t)]({\textsf{P}}). \end{aligned}$$

We conclude from the LT definition of the MABC the following:

$$\begin{aligned}{}&\frac{M(\vartheta )}{1-\vartheta }\ \frac{{\textsf{P}}^{\vartheta } {\mathscr {L}}[R(t);{\textsf{P}}]- {\textsf{P}}^{\vartheta -1} R(0)}{{\textsf{P}}^{\vartheta }+\mu _{\vartheta }}=\rho ^{1-\vartheta } {\mathscr {L}}[-\alpha R(t)+\beta W(t)]({\textsf{P}}), \\&\frac{M(\vartheta )}{1-\vartheta }\ \frac{{\textsf{P}}^{\vartheta } {\mathscr {L}}[W(t); {\textsf{P}}]- {\textsf{P}}^{\vartheta -1} W(0)}{ {\textsf{P}}^{\vartheta }+\mu _{\vartheta }}=\rho ^{1-\vartheta } {\mathscr {L}}[\alpha R(t)-(\beta +\delta ) W(t)]({\textsf{P}}). \end{aligned}$$

If we reorder the inequalities shown above, then

$$\begin{aligned} {\mathscr {L}}[R(t);{\textsf{P}}]= \frac{R(0)}{{\textsf{P}}} + \frac{\rho ^{1-\vartheta } (1-\vartheta )}{M(\vartheta )} \, \Big ( 1+ \frac{\mu _{\vartheta }}{{\textsf{P}}^{\vartheta }}\Big ) \, {\mathscr {L}}[-\alpha R(t)+\beta W(t)], \\ {\mathscr {L}}[W(t); {\textsf{P}}]= \frac{W(0)}{{\textsf{P}}}+\frac{\rho ^{1-\vartheta } (1-\vartheta )}{M(\vartheta )} \, \Big ( 1+ \frac{\mu _{\vartheta }}{{\textsf{P}}^{\vartheta }}\Big ) \, {\mathscr {L}}[\alpha R(t)-(\beta +\delta ) W(t)]. \end{aligned}$$

We get

9 $$\begin{aligned} \left. \begin{aligned}{}&R_{n+1}(t)=R_{n}(0) + {\mathscr {L}}^{-1} \bigg [ \frac{\rho ^{1-\vartheta } (1-\vartheta )}{M(\vartheta )} \, \Big ( 1+ \frac{\mu _{\vartheta }}{{\textsf{P}}^{\vartheta }}\Big ) \, {\mathscr {L}}[-\alpha R_{n}(t)+\beta \, W_{n}(t)] \bigg ], \\&W_{n+1}(t)=W_{n}(0) + {\mathscr {L}}^{-1} \bigg [ \frac{\rho ^{1-\vartheta } (1-\vartheta )}{M(\vartheta )} \, \Big ( 1+ \frac{\mu _{\vartheta }}{{\textsf{P}}^{\vartheta }}\Big ) \, {\mathscr {L}}[\alpha R_{n}(t)-(\beta +\delta ) W_{n}(t)] \bigg ]. \ \ \ \ \ \end{aligned} \right\} \end{aligned}$$

The system’s approximate solution (2) is as follows:

$$\begin{aligned} R(t)=lim_{n\rightarrow \infty } R_{n}(t), \\ W(t)=lim_{n\rightarrow \infty } W_{n}(t). \end{aligned}$$

### Evaluation of the iteration method’s stability

Let us consider the recursive method $p_{n+1}=\chi ({\mathscr {T}}, R_{n})$ and a self-map ${\mathscr {T}}$ on Banach space $(H, \Vert .\Vert )$. Presume that $\zeta ({\mathscr {T}})\ne \phi$ is the fixed point set of ${\mathscr {T}}$ and which is $lim_{n\rightarrow \infty }p_{n}=p\in \zeta ({\mathscr {T}})$. Presume that $\{t_{n}\}\subset \zeta$ and $j_{n}=\Vert t_{n+1}-\chi ({\mathscr {T}},t_{n})\Vert$. If $lim_{n\rightarrow \infty }j_{n}=0 \implies lim_{n\rightarrow \infty }t_{n}=p$, then the iterative process $p_{n+1}=\chi ({\mathscr {T}},p_{n})$ is ${\mathscr {T}}$-stable. Presume there is an upper limit for our sequence $\{t_{n}\}$. If all of these circumstances are fulfilled for Picard’s iteration $p_{n+1}={\mathscr {T}} p_{n}$, then $p_{n+1}={\mathscr {T}} p_{n}$ is ${\mathscr {T}}$-stable.

#### Theorem 5.1

Wang et al.^28^ Let ${\mathscr {T}}$ be a self-map on Banach space $(H, \Vert .\Vert )$, satisfying $\Vert {\mathscr {T}}_{y}-{\mathscr {T}}_{z}\Vert \le A\Vert y-{\mathscr {T}}_{y}\Vert +a\Vert y-z\Vert$ for all $y, z\in H$ where $A\ge 0$ and $0\le a <1$. Assume that ${\mathscr {T}}$ is Picard ${\mathscr {T}}$-stable.

As per Eq. (9), the Human-Liver (2) fractional model is related to the subsequent iterative formula. Now take a look at the following theorem.

#### Theorem 5.2

Assume that ${\mathscr {T}}$ is a self-map defined as follows:

$$\begin{aligned}{}&{\mathscr {T}}(R_{n}(t))=R_{n+1}(t) \\&= R_{n}(t)+ {\mathscr {L}}^{-1} \bigg [ \frac{\rho ^{1-\vartheta } (1-\vartheta )}{M(\vartheta )} \, \Big ( 1+ \frac{\mu _{\vartheta }}{{\textsf{P}}^{\vartheta }}\Big ) \, {\mathscr {L}}[-\alpha R_{n}(t)+\beta \, W_{n}(t)] \bigg ], \\&{\mathscr {T}}(W_{n}(t))=W_{n+1}(t) \\&=W_{n}(t)+ {\mathscr {L}}^{-1} \bigg [ \frac{\rho ^{1-\vartheta } (1-\vartheta )}{M(\vartheta )} \, \Big ( 1+ \frac{\mu _{\vartheta }}{{\textsf{P}}^{\vartheta }}\Big ) \, {\mathscr {L}}[\alpha R_{n}(t)-(\beta +\delta ) W_{n}(t)] \bigg ]. \end{aligned}$$

If the following conditions are met, this iterative recursive is ${\mathscr {T}}$-stable in $L^{1}(a,b)$:

$$\begin{aligned}{}&(1-\alpha g_{1}(\vartheta )+\beta g_{2}(\vartheta ))<1, \\&(1+\alpha g_{3}(\vartheta )-(\beta +\delta )g_{4}(\vartheta ))<1. \end{aligned}$$

#### Proof

To demonstrate that ${\mathscr {T}}$ has a fixed point, For $(k, l)\in N\times N$, we compute the following inequalities:

$$\begin{aligned}{}&{\mathscr {T}}(R_{k}(t))-{\mathscr {T}} (R_{l}(t)) \\&=(R_{k}(t)-R_{l}(t))+ {\mathscr {L}}^{-1} \bigg [ \frac{\rho ^{1-\vartheta } (1-\vartheta )}{M(\vartheta )} \, \Big ( 1+ \frac{\mu _{\vartheta }}{{\textsf{P}}^{\vartheta }}\Big ) \, {\mathscr {L}} \big [-\alpha (R_{k}(t)-R_{l}(t)) + \beta (W_{k}(t)- W_{l}(t)) \big ]\bigg ]. \end{aligned}$$

Applying the norm on both halves, we get

10 $$\begin{gathered} \left\| { {\mathscr{T}}(R_{k} (t)) - {\mathscr{T}}(R_{l} (t))} \right\| \le \left\| {(R_{k} (t) - R_{l} (t))} \right\| \hfill \\ + {\mathscr{L}}^{{ - 1}} \left[ {\frac{{\rho ^{{1 - \vartheta }} (1 - \vartheta )}}{{M(\vartheta )}}(1 + \frac{{\mu _{\vartheta } }}{{\textsf{P}^{\vartheta } }}) {\mathscr{L}}\left[ {\left\| { - \alpha (R_{k} (t) - R_{l} (t))} \right\| + \left\| {\beta (W_{k} (t) - W_{l} (t))} \right\|} \right]} \right]. \hfill \\ \end{gathered}$$

Since the roles in the solution are the same, we may think about

11 $$\begin{aligned} \Vert R_{k}(t)-R_{l}(t)\Vert \cong \Vert W_{k}(t)-W_{l}(t)\Vert \end{aligned}$$

From Eqs. (10) and (11), we get

12 $$\begin{gathered} \left\| { {\mathscr{T}}(R_{k} (t)) - {\mathscr{T}}(R_{l} (t))} \right\| \le \left\| {(R_{k} (t) - R_{l} (t))} \right\| \hfill \\ + {\mathscr{L}}^{{ - 1}} \left[ {\frac{{\rho ^{{1 - \vartheta }} (1 - \vartheta )}}{{M(\vartheta )}}(1 + \frac{{\mu _{\vartheta } }}{{\textsf{P}^{\vartheta } }}) {\mathscr{L}}\left[ {\left\| { - \alpha (R_{k} (t) - R_{l} (t))} \right\| + \left\| {\beta (R_{k} (t) - R_{l} (t))} \right\|} \right]} \right] \hfill \\ \le \left[ {1 - \alpha g_{1} (\vartheta ) + \beta g_{2} (\vartheta )} \right]\left\| {R_{k} (t) - R_{l} (t)} \right\|. \hfill \\ \end{gathered}$$

Where $g_{k}$ are functions from

$$\begin{aligned} {\mathscr {L}}^{-1}\bigg [\frac{\rho ^{1-\vartheta } (1-\vartheta )}{M(\vartheta )} \, \Big ( 1+ \frac{\mu _{\vartheta }}{{\textsf{P}}^{\vartheta }}\Big ) {\mathscr {L}} [\star ]\bigg ]. \end{aligned}$$

Similarly, we will obtain

13 $$\begin{aligned} \Vert {\mathscr {T}} (W_{k}(t))- {\mathscr {T}} (W_{l}(t))\Vert \le \big [1+\alpha g_{3}(\vartheta )-(\beta +\delta )g_{4}(\vartheta )\big ] \Vert W_{k}(t)-W_{l}(t)\Vert . \end{aligned}$$

Where

$$\begin{aligned}{}&(1-\alpha g_{1}(\vartheta )+\beta g_{2}(\vartheta ))<1, \\&(1+\alpha g_{3}(\vartheta )-(\beta +\delta )g_{4}(\vartheta ))<1. \end{aligned}$$

Thus ${\mathscr {T}}$ is a fixed point of self-mapping. Additionally, we demonstrate that ${\mathscr {T}}$ meets the requirements in Theorem (5.1). Consider that (12), (13) hold, we suppose

$$A=(0, 0),$$

$$\begin{aligned} a= \left\{ \begin{aligned}{}&(1-\alpha g_{1}(\vartheta )+\beta g_{2}(\vartheta )), \\&(1+\alpha g_{3}(\vartheta )-(\beta +\delta )g_{4}(\vartheta )). \end{aligned} \right. \end{aligned}$$

Consequently, all of Theorem (5.1)’s conditions have been fulfilled, and the proof is finished. $\square$

## Numerical implementation

In this part, we apply HATM (homotopy analysis transform method) to build the model (2) reasonably accurately. It should be noted that HATM is a well-developed improvement of the traditional LT method^29^ and HAM^30^. To keep the homogeneity and stability assessment of the proposed model accurate and precise, the stability and structure of the model equations under different conditions have been thoroughly considered. To solve the model (2) using HATM, we first apply LT as shown below:

$$\begin{aligned}{}&{\mathscr {L}} \Big [\frac{1}{\rho ^{1-\vartheta }} \, {}^{{\textit{MABC}}} {\mathscr {D}}_{t}^{\vartheta } R(t)\Big ]({\textsf{P}})= {\mathscr {L}}[-\alpha R(t)+\beta W(t)]({\textsf{P}}), \\&{\mathscr {L}} \Big [\frac{1}{\rho ^{1-\vartheta }} \, {}^{{\textit{MABC}}}{\mathscr {D}}_{t}^{\vartheta } W(t)\Big ]({\textsf{P}})= {\mathscr {L}}[\alpha R(t)-(\beta +\delta ) W(t)]({\textsf{P}}), \end{aligned}$$

which results in

$$\begin{aligned}{}&\frac{M(\vartheta )}{1-\vartheta }\ \frac{{\textsf{P}}^{\vartheta } {\mathscr {L}}[R(t); {\textsf{P}}]- {\textsf{P}}^{\vartheta -1} R(0)}{{\textsf{P}}^{\vartheta }+\frac{\vartheta }{1-\vartheta }}=\rho ^{1-\vartheta } {\mathscr {L}}[-\alpha R(t)+\beta W(t)]({\textsf{P}}), \\&\frac{M(\vartheta )}{1-\vartheta }\ \frac{ {\textsf{P}}^{\vartheta } {\mathscr {L}}[W(t); {\textsf{P}}]- {\textsf{P}}^{\vartheta -1} W(0)}{ {\textsf{P}}^{\vartheta }+\frac{\vartheta }{1-\vartheta }}=\rho ^{1-\vartheta } {\mathscr {L}}[\alpha R(t)-(\beta +\delta ) W(t)]({\textsf{P}}). \end{aligned}$$

Then we have

14 $$\begin{aligned} \left. \begin{aligned}{}&{\mathscr {L}}[R(t); {\textsf{P}}]-\frac{R(0)}{{\textsf{P}}}-\frac{\rho ^{1-\vartheta }}{M(\vartheta )} \, \Big ( 1-\vartheta + \frac{\vartheta }{ {\textsf{P}}^{\vartheta }} \Big ) {\mathscr {L}} [-\alpha R(t)+\beta W(t)]({\textsf{P}})=0, \\&{\mathscr {L}}[W(t); {\textsf{P}}]-\frac{W(0)}{{\textsf{P}}}-\frac{\rho ^{1-\vartheta }}{M(\vartheta )} \, \Big ( 1-\vartheta + \frac{\vartheta }{ {\textsf{P}}^{\vartheta }} \Big ) {\mathscr {L}}[\alpha R(t)-(\beta +\delta ) W(t)]({\textsf{P}})=0. \end{aligned} \right\} \end{aligned}$$

Using the homotopy method, we define

$$\begin{aligned}{}&N _{1}(\varPhi _{1}(t; r), \varPhi _{2}(t; r))={\mathscr {L}}[-\alpha \, \varPhi _{1}(t; r)+\beta \, \varPhi _{2}(t; r)], \\&N _{2}(\varPhi _{1}(t; r), \varPhi _{2}(t; r))={\mathscr {L}}[\alpha \, \varPhi _{1}(t; r)-(\beta +\delta ) \, \varPhi _{2}(t; r)]. \end{aligned}$$

Consequently, the deformation equations become

$$\begin{aligned}{}&(1-r){\mathscr {L}}[\varPhi _{1}(t;r)-R_{0}(t)]=rhH(t) N _{1}(\varPhi _{1}, \varPhi _{2}), \\&(1-r){\mathscr {L}}[\varPhi _{2}(t;r)-W_{0}(t)]=rhH(t) N _{2}(\varPhi _{1}, \varPhi _{2}), \end{aligned}$$

where $r\in [0,1]$ denotes an embedding parameter, $\varPhi _{j}(t;r), \ j=0,1$ are unknown functions, $R_{0}, W_{0}$ are preliminary estimates, $h \ne 0$ is an auxiliary parameter, ${\mathscr {L}}[.]$ is the Laplace operator, and $H(t)\ne 0$ is an auxiliary function.

Certainly, for $r=0$ and $r=1$, we get

$$\begin{aligned}{}&\varPhi _{1}(t;0)= R_{0}(t), \ \ \ \ \varPhi _{1}(t;1)=R(t), \\&\varPhi _{2}(t;0)= W_{0}(t), \ \ \ \ \varPhi _{2}(t;1)=W(t). \end{aligned}$$

As a result, increasing *r* from 0 to 1 changes the solution $(\varPhi _{1}(t;r), \varPhi _{2}(t;r))$ from $(R_{0}(t), W_{0}(t))$ to (*R*(*t*), *W*(*t*)). Here, we expand $\varPhi _{j}(t;r)\ (j=1,2)$ in the Taylor series concerning *r*.

This technique produces

$$\begin{aligned}{}&\varPhi _{1}(t;r)=R_{0}+\sum _{\eta =1}^{\infty }R_{\eta }(t)r^{\eta }, \\&\varPhi _{2}(t;r)=W_{0}+\sum _{\eta =1}^{\infty }W_{\eta }(t)r^{\eta }, \end{aligned}$$

where

15 $$\begin{aligned} R_{\eta } (t) = & \frac{1}{{\eta !}}\frac{{\partial ^{\eta } \varPhi _{1} (t;r)}}{{\partial r^{\eta } }}|_{{r = 0}} , \\ W_{\eta } (t) = & \frac{1}{{\eta !}}\frac{{\partial ^{\eta } \varPhi _{2} (t;r)}}{{\partial r^{\eta } }}|_{{r = 0}} . \\ \end{aligned}$$

According to Liao^30^, the series (15) converges at $r=1$ if *H*(*t*), *h*, and the starting estimates are suitably selected. Thus, we obtain

$$\begin{aligned}{}&R(t)=R(0)+\sum _{\eta =1}^{\infty }R_{\eta }(t), \\&W(t)=W(0)+\sum _{\eta =1}^{\infty }W_{\eta }(t). \end{aligned}$$

Additionally, the $\eta$th order deformation equation may be expressed as

16 $$\begin{aligned} {\mathscr{L}}\left[ {R_{\eta } (t) - \psi _{\eta } R_{{\eta - 1}} (t)} \right] = & hH(t)T_{{1,\eta }} (R_{{\eta - 1}} (t),W_{{\eta - 1}} (t)), \\ {\mathscr{L}}\left[ {W_{\eta } (t) - \psi _{\eta } W_{{\eta - 1}} (t)} \right] = & hH(t)T_{{2,\eta }} (R_{{\eta - 1}} (t),W_{{\eta - 1}} (t)), \\ \end{aligned}$$

where

17 $$\begin{aligned} \left. \begin{aligned}{}&T_{1, \eta }(R_{\eta -1}(t), W_{\eta -1}(t))={\mathscr {L}}[R_{\eta -1}(t)]-\frac{R(0)}{{\textsf{P}}}(1-\psi _{\eta }) \\&\ \ \ \ \ \ \ \ \ \ \ \ \ \ \ \ -\frac{\rho ^{1-\vartheta }}{M(\vartheta )} \, \Big ( 1-\vartheta + \frac{\vartheta }{ {\textsf{P}}^{\vartheta }} \Big ) {\mathscr {L}}[-\alpha R_{\eta -1}(t)+\beta W_{\eta -1}(t)], \\&T_{2, \eta }(R_{\eta -1}(t), W_{\eta -1}(t))={\mathscr {L}}[W_{\eta -1}(t)]-\frac{W(0)}{{\textsf{P}}}(1-\psi _{\eta }) \\&\ \ \ \ \ \ \ \ \ \ \ \ \ \ \ \ \ -\frac{\rho ^{1-\vartheta }}{M(\vartheta )} \, \Big ( 1-\vartheta + \frac{\vartheta }{ {\textsf{P}}^{\vartheta }} \Big ) {\mathscr {L}}[\alpha R_{\eta -1}(t)-(\beta +\delta ) W_{\eta -1}(t)], \end{aligned} \right\} \end{aligned}$$

and

$$\begin{aligned} \psi _{\eta }= \left\{ \begin{aligned}{}&0,\ \ \ \ \ \ \eta \le 1,\\&1,\ \ \ \ \ \ \eta >1. \end{aligned} \right. \end{aligned}$$

Using the inverse LT to Eq. (16), we obtain

$$\begin{aligned}{}&R_{\eta }(t)=\psi _{\eta }R_{\eta -1}(t)+h H(t) {\mathscr {L}}^{-1}[T_{1, \eta }(R_{\eta -1}(t), W_{\eta -1}(t))], \\&W_{\eta }(t)=\psi _{\eta }W_{\eta -1}(t)+h H(t) {\mathscr {L}}^{-1}[T_{2, \eta }(R_{\eta -1}(t), W_{\eta -1}(t))]. \end{aligned}$$

Through the solution of these equations for various values of $\eta =1,2,3,\cdots$, we get to

$$\begin{aligned}{}&R_{1}(t)= \alpha \,h H(t)\, R_{0}(t) \frac{\rho ^{1-\vartheta }}{M(\vartheta )}\Big (1-\vartheta +\frac{t^{\vartheta }}{\Gamma (\vartheta )}\Big ), \\&W_{1}(t)=-\alpha \,h H(t) \, R_{0}(t) \frac{\rho ^{1-\vartheta }}{M(\vartheta )}\Big (1-\vartheta +\frac{t^{\vartheta }}{\Gamma (\vartheta )}\Big ). \end{aligned}$$

Ultimately, the outcomes of system (2) are achieved as follows:

$$\begin{aligned} R(t)=R_{0}(t)+R_{1}(t)+R_{2}(t)+\cdots \end{aligned}$$

18 $$\begin{gathered} R(t) = R_{0} (t) + hH(t)(2 + hH(t))R_{0} (t){\mkern 1mu} \alpha \frac{{\rho ^{{1 - \vartheta }} }}{{M(\vartheta )}}\left( {1 - \vartheta + \frac{{t^{\vartheta } }}{{\Gamma (\vartheta )}}} \right) + (\alpha + \beta )R_{0} (t)\alpha h^{2} H^{2} \hfill \\ \frac{{\rho ^{{2 - 2\vartheta }} }}{{(M(\vartheta ))^{2} }}\left( {(1 - \vartheta )^{2} + \frac{{2(1 - \vartheta )t^{\vartheta } }}{{\Gamma (\vartheta )}} + \frac{{\vartheta ^{2} {\mkern 1mu} t^{{2\vartheta }} }}{{\Gamma (2\vartheta + 1)}}} \right), \hfill \\ \end{gathered}$$

and

$$\begin{aligned} W(t)=W_{0}(t)+W_{1}(t)+W_{2}(t)+\cdots \end{aligned}$$

19 $$\begin{gathered} W(t) = - hH(t)(2 + hH(t))R_{0} (t)\alpha \frac{{\rho ^{{1 - \vartheta }} }}{{M(\vartheta )}}\left( {1 - \vartheta + \frac{{t^{\vartheta } }}{{\Gamma (\vartheta )}}} \right) - (\alpha + \beta + \delta )R_{0} (t)\alpha h^{2} H^{2} \hfill \\ \frac{{\rho ^{{2 - 2\vartheta }} }}{{(M(\vartheta ))^{2} }}\left( {(1 - \vartheta )^{2} + \frac{{2(1 - \vartheta )t^{\vartheta } }}{{\Gamma (\vartheta )}} + \frac{{\vartheta ^{2} t^{{2\vartheta }} }}{{\Gamma (2\vartheta + 1)}}} \right). \hfill \\ \end{gathered}$$

Derivative of *R*(*t*) and *W*(*t*) (with $H=1,t=0, \text {and} \, \vartheta =1$) as follows:

20 $$\begin{aligned} R^{\prime } (0) = & (2 + h)hR_{0} (t)\alpha , \\ R^{{\prime \prime }} (0) = & (\alpha + \beta )h^{2} R_{0} (t)\alpha , \\ R^{{\prime \prime \prime }} (0) = & 0, \\ \end{aligned}$$

and

21 $$\begin{aligned} W^{\prime } (0) = & - (2 + h)hR_{0} (t){\mkern 1mu} \alpha , \\ W^{{\prime \prime }} (0) = & - (\alpha + \beta + \delta )h^{2} R_{0} (t)\alpha , \\ W^{{\prime \prime \prime }} (0) = & 0. \\ \end{aligned}$$

### Convergency of HATM of FDEs

In this part, we examine the convergence of HATM by introducing and demonstrating the following theorem.

#### Theorem 6.1

*R*(*t*) and *W*(*t*) have the precise solution for the system (14) if they fulfill the following conditions:

(i) $\{R(t), W(t)\}\in L(\mathbb{R}^{+})$ are engendered by the $\eta$th order deformation (16),
(ii) $\sum _{\eta =0}^{\infty } R_{\eta }(t)$, and $\sum _{\eta =0}^{\infty } W_{\eta }(t)$ be uniformly convergent (UC) to *R*(*t*), and *W*(*t*), respectively,
(iii) let $\sum _{\eta =0}^{\infty } {}^{MABC}D_{t}^{\vartheta } R_{\eta }(t)$ and $\sum _{\eta =0}^{\infty } {}^{MABC}D_{t}^{\vartheta } W_{\eta }(t)$ be convergent series.

#### Proof

By presuming that $\sum _{\eta =0}^{\infty } R_{\eta }(t)$ is UC to *R*(*t*), we may plainly say

$lim_{\eta \rightarrow \infty } R_{\eta }(t)=0, \ t\in \mathbb{R}^{+}.$ Laplace being a linear operator, we possess

22 $$\begin{aligned} \begin{aligned}{}&\sum _{\eta =1}^{\mathfrak {i}} {\mathscr {L}}[R_{\eta }(t)-\psi _{\eta }R_{\eta -1}(t)] =\sum _{\eta =1}^{\mathfrak {i}} [{\mathscr {L}} R_{\eta }(t)-\psi _{\eta } {\mathscr {L}} R_{\eta -1}(t)] \\&={\mathscr {L}}(R_{1}(t))+({\mathscr {L}}R_{2}(t)-{\mathscr {L}}R_{1}(t))+...+({\mathscr {L}}R_{\mathfrak {i}}(t)-{\mathscr {L}}R_{\mathfrak {i}-1}(t))= {\mathscr {L}} R_{\mathfrak {i}}(t). \end{aligned} \end{aligned}$$

Thus, from (6.1) and (22) we derive

$$\begin{aligned} \sum _{\eta =1}^{\mathfrak {i}} {\mathscr {L}}[R_{\eta }(t)-\psi _{\eta }R_{\eta -1}(t)] =lim_{\mathfrak {i}\rightarrow \infty }{\mathscr {L}}R_{\mathfrak {i}}(t) = {\mathscr {L}}\big (lim_{\mathfrak {i}\rightarrow \infty }R_{\mathfrak {i}}(t)\big )=0. \end{aligned}$$

Hence,

$$\begin{aligned} hH\sum _{\eta =1}^{\infty } T_{1,\eta }\big (R_{\eta -1}(t), W_{\eta -1}(t)\big )=\sum _{\eta =1}^{\infty }{\mathscr {L}}\big [R_{\eta }(t)-R_{\eta -1}(t)\big ]=0. \end{aligned}$$

Since $h\ne 0, H\ne 0$, this yields $\sum _{\eta =1}^{\infty } T_{1,\eta }\big (R_{\eta -1}(t), W_{\eta -1}(t)\big )=0$. Likewise, we can demonstrate

$$\begin{aligned} \sum _{\eta =1}^{\infty } T_{2,\eta }\big (R_{\eta -1}(t), W_{\eta -1}(t)\big )=0. \end{aligned}$$

Now, from (17) we obtain

$$\begin{aligned} \begin{aligned}0=&\sum _{\eta =1}^{\infty }\left [{\mathscr {L}}[R_{\eta -1}(t)]-\frac{R(0)}{s}(1-\psi _{\eta })\right. \\&\left.-\frac{\rho ^{1-\vartheta }(1-\vartheta )\left (s^{\vartheta }+\frac{\vartheta }{1-\vartheta }\right )}{s^{\vartheta }\ \ M(\vartheta )} {\mathscr {L}}[-\alpha R_{\eta -1}(t)+\beta W_{\eta -1}(t)]\right ] \\=&{\mathscr {L}} \left (\sum _{\eta =1}^{\infty }R_{\eta -1}(t)\right )-\frac{R(0)}{s} \sum _{\eta =1}^{\infty } (1-\psi _{\eta }) \\&-\frac{\rho ^{1-\vartheta }(1-\vartheta )\left (s^{\vartheta }+\frac{\vartheta }{1-\vartheta }\right )}{s^{\vartheta }\ \ M(\vartheta )} {\mathscr {L}}\left [\sum _{\eta =1}^{\infty } [-\alpha R_{\eta -1}(t)+\beta W_{\eta -1}(t)]\right ] \\&={\mathscr {L}} \left [ R(t)\right ]-\frac{R(0)}{s} -\frac{\rho ^{1-\vartheta }(1-\vartheta )\left (s^{\vartheta }+\frac{\vartheta }{1-\vartheta }\right )}{s^{\vartheta }\ \ M(\vartheta )} {\mathscr {L}}\left [ -\alpha R(t)+\beta W(t)\right ]. \end{aligned} \end{aligned}$$

Therefor *R*(*t*) is the precise solution of system (14). Likewise, we can demonstrate that *W*(*t*) are the exact solution of the system(14), and the proof is finished. $\square$

## Discussion and results

This part presents the HATM to give a numerical simulation for the Human-Liver model (2). The BSP model is solved using parameters selected from Table 1 for the computational simulation.

**Table 1:** The variables and parameter values of the model (Figure 1)^17,20^.

Variable/parameter	Description	Value
*R*(*t*)	The concentration of BSP in the blood at any time *t*	$R(0)=250$
*W*(*t*)	The concentration of BSP in liver at any time *t*	$W(0)=0$
$\alpha$	The rate of BSP transfer from blood to liver	0.054736
$\beta$	The rate of BSP elimination from the liver	0.0152704
$\delta$	The rate at which BSP flows back into the blood from the liver	0.0093906

In the beginning, the effect of *h* on the convergence of the series solutions derived by the HATM is explored. This parameter sets the convergence zone and rate of convergence for the HATM approximations. We depict the *h*-curves for the approximate solutions to Eq. (2) to understand the influence of *h*.

**Figure 2:**
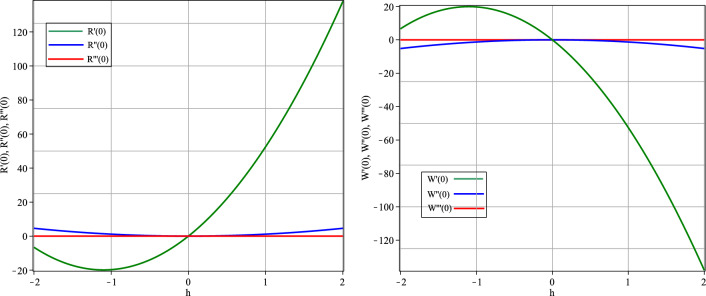
The *h*-curves for *R*(*t*) and *W*(*t*)’s HATM solutions.

Figure 2 shows plots of *h* with $R'(0), R''(0), R'''(0)$ and $W'(0), W''(0), W'''(0)$ for $\vartheta =1$. Based on the figure, we pick the horizontal line parallel to the *h*-axis as the convergence zone for the approximations, therefore the convergence of the approach is assured for $-1.5\le h \le -0.5$. As a result, the middle point of this range, $h=-1$, is an excellent choice for *h* at which the numerical solution converges^31^.

**Figure 3:**
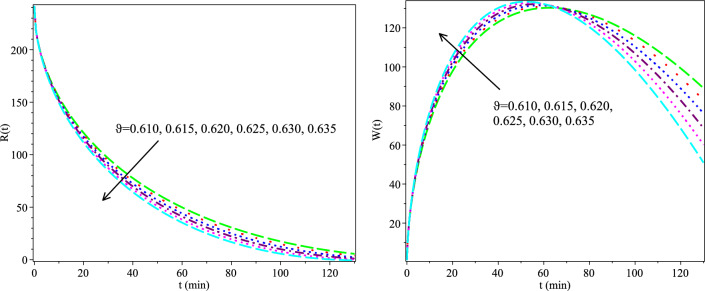
Graphs of the HATM solutions for $h=-1,$ and $H=1$.

**Figure 4:**
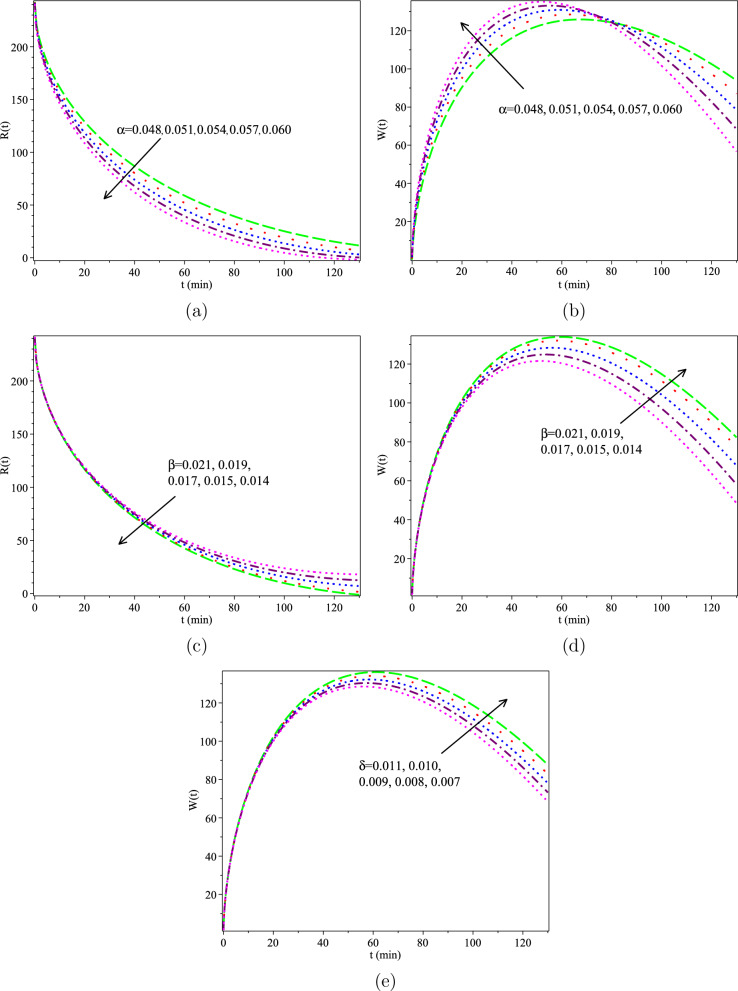
For various values of $\alpha , \beta$, and $\delta$, the graphs of *R*(*t*) and *W*(*t*) are (**a**) and (**b**), (**c**) and (**d**), and (**e**), respectively.

The Adomian Decomposition Method (ADM)^32^, the CFFD^20^, and the MABC findings are compared in Tables 2 and 3 using clinical data collected by Evzen Hrncif in 1985^17^ with $h=-1, \vartheta =1 \ \text {and}\ H=1$. As shown, the MABC offers more accurate findings than the ADM and HATM in terms of actual experimental observations. The MABC solutions are then calculated for values of $\vartheta = 0.610, 0.615, 0.620, 0.625, 0.630, 0.635, \ h =-1, \ \text {and} \ H = 1$. The findings are shown in Figure 3, demonstrating that as $\vartheta \rightarrow 1$, the approximate solutions converge to the traditional integer answers. The MABC also performs well for $\vartheta$’s around 1 and lesser ones. Figure 4 shows the measured values of BSP in blood and bile, corresponding to different values of the unknown parameters $\alpha , \beta$, and $\delta$.

**Table 2:** Comparison between Real data, ADM, HATM, and MABC for *R*(*t*) with fractional order $\vartheta =0.0630$.

Time(t)	0	3	5	10	20	30	43
Real data^17^	250	221	184	141	98	80	64
CFFD^20^	250	212.80	191.12	149.60	100.45	82.93	62.34
ADM^32^	250	212.76	192.83	153.08	85.19	70.91	55.81
MABC	250	198.97	180.84	149.06	110.12	84.27	61.05

**Table 3:** Comparison between Real data, ADM, HATM, and MABC for *W*(*t*) with fractional order $\vartheta =0.0630$.

Time(t)	0	5	10	20	30
Real data^17,20^	0	65.80	106.50	141.50	148.50
CFFD^20^	0	59.87	94.13	136.17	143.92
ADM^32^	0	59.14	92.09	134.23	143.92
MABC	0	53.12	77.31	105.47	121.80

## Conclusion

In this study, we propose a novel MABC fractional derivative model of the human liver using a nonsingular kernel that not only contributes to its mathematical robustness but also aligns with the biological complexity of the human liver. A parameter adjustment has been used to prevent dimensional mismatching. In addition, the iteration method and fixed point theory were used to look into the possibility of a single solution. A fresh and potent numerical approach was also offered to execute the proposed model in an acceptable, accurate way. The recommended method’s stability was also examined and assessed. Additionally, certain numerical tests in Tables 2 and 3 and Figure 3 were used to confirm the effectiveness of the new strategy. Figure 3 also demonstrates the viability of the suggested fractional calculus modeling. Compared with earlier studies, the specific order of the novel fractional models follows reality better than the classic integer solutions. This study may not be entirely responsible for diverse biological characteristics between different patients. The model’s projection may be limited for a broader population, and its forecasting accuracy may vary in individuals. For example, according to the perspective of quantifying BSP, Figure 4 is related to various values of unknown factors $\alpha , \beta$, and $\delta$. These parameters may be crucial in the pharmaceutical sector. According to this study, the estimate of human liver damage is known earlier than the integer order, which is necessary for much better treatment. Future research should focus on refining the model by incorporating more biological realism, considering personal variability, and conducting complete verification in diverse clinical scenarios.

### Ethical approval

No personal or sensitive information is disclosed or compromised.

## Data Availability

All data generated or analyzed during this study are included in this published article^17,20^. Further, we declare that no human data or participants have been involved in the study.
